# Glofitamab therapy for diffuse large B cell lymphoma: latest updates from the 2022 ASH Annual Meeting

**DOI:** 10.1186/s13045-023-01420-w

**Published:** 2023-03-10

**Authors:** Chaoyu Wang, Yao Liu

**Affiliations:** Department of Hematology, Chongqing University Cancer Hospital, Chongqing Key Laboratory of Translational Research for Cancer Metastasis and Individualized Treatment, Chongqing, 400030 China

## Abstract

Over one-third of B cell lymphomas are not effectively treated by R-CHOP chemotherapy. When lymphoma relapses or is refractory, the prognosis becomes very poor. Due to this fact, there is an urgent and clear requirement for a more effective, novel treatment option. Glofitamab is a CD20xCD3 T-cell-engaging, bispecific antibody capable of recruiting T cells to tumor cells. We have summarized several of the latest reports on glofitamab use in B cell lymphoma therapy from the 2022 ASH Annual Meeting (ASH2022).

**To the editor**

The prognosis for patients with relapsed or refractory (R/R) B-cell lymphoma is generally poor. New therapeutic methods are urgently required within clinical settings. Glofitamab is a CD20xCD3 T-cell-engaging bispecific antibody with a 2:1 (CD20:CD3) configuration. It confers high-avidity bivalent binding to CD20 on B cells, and recruits T cells directly to tumor cells [1, 2]. We have summarized several of the latest reports on glofitamab in B cell lymphoma therapy from the 2022 ASH Annual Meeting (ASH2022).

## Glofitamab for newly diagnosed diffuse large B cell lymphoma (DLBCL)

A phase Ib study [3] assessed the role of the glofitamab inclusion in an R-CHOP regimen in patients with newly diagnosed, untreated DLBCL. In total, 56 patients were enrolled. The median age of the patients was 68 years (from 21 to 84 years of age), and 96.4% presented with advanced stage B cell lymphoma (III/IV). Among the 46 patients who were evaluable, the overall response rate (ORR) and complete response (CR) rate were 93.5% and 76.1%, respectively. With respect to the safety of this treatment, grade ≥ 3 adverse events (AEs) related to glofitamab were observed in 13 (23.2%) patients. No reports of grade 3–5 cytokine release syndrome (CRS) events were presented.

## Glofitamab for R/R B cell lymphoma

A phase I clinical trial study [4] reported on two bispecific monoclonal antibodies (RO7227166 and glofitamab) in R/R B-cell lymphoma. RO7227166 is a bispecific monoclonal antibody which targets CD19 and 4-1BB. In the presence of a T-cell receptor signal and strict dependence upon CD19 crosslinking, RO7227166 provides a strong stimulation to T-cells via 4-1BB agonism. 4-1BB upregulation was stimulated on immune cells by glofitamab. A total of 71 patients were enrolled in the trial. 44.3% of patients had advanced stage B-cell lymphoma, while 27.1% had received prior CAR-T therapy. The median number of prior therapeutic approaches was 3 (ranging from 1 to 7). In DLBCL, the ORR and CR were 67% and 39%, respectively. However, the ORR and CR were 91% and 73% in FL patients (Table 1). Grade ≥ 3 AEs were observed to occur in 57.2% patients (Table 2).

**Table 1:** Updates on glofitamab therapy for B cell lymphoma patients

Author	Study type	Regimen	Patients	ORR	CR	Survival
Max S. Topp	Phase Ib	Glofitamab combined with R-CHOP	Newly diagnosed untreated DLBCL, N = 56	93.5%	76.1%	–
Martin Hutchings	Phase I	RO7227166 combined with glofitamab	R/R B-cell lymphoma, N = 71 (46 DLBCL, 24 FL, 1 MZL)	DLBCL:67% FL:91%	DLBCL:39% FL:73%	–
Anna Dodero	Retrospective	Glofitamab	R/R large B-cell lymphomas, N = 18	68%	38%	1-year OS: 44%
Martin Hutchings	Retrospective	Glofitamab	R/R large B-cell lymphoma, N = 61 (41 DLBCL, 18 tFL, 1 HGBCL, 1 PMBCL)	84%	56%	–
Burhan Ferhanoglu	Retrospective (compassionate use)	Glofitamab	R/R large B-cell lymphoma, N = 42 (2 tFL, 40 DLBCL),	28.5%	19%	Median OS: 7 months

*DLBCL* diffuse large B cell lymphoma, *tFL* transformed follicular lymphomas, *PMBCL* primary mediastinal B-Cell lymphoma, *HGBCL* high-grade B-cell lymphoma, *MZL* marginal zone lymphoma, *R/R* relapsed/refractory, *CR* complete response, *ORR* overall response rate, *PFS* progression free survival, *OS* overall survival

**Table 2:** Reported adverse events from glofitamab therapy

Author	Study	Regimen	Grade ≥ 3 AEs	Grade 1–2 CRS	Grade 3–5 CRS
Max S. Topp	Phase Ib	Glofitamab combined with R-CHOP	71.4%	10.7%	0
Martin Hutchings	Phase I	RO7227166 combined with glofitamab	57.2%	54.3%	0
Anna Dodero	Retrospective	Glofitamab	–	–	–
Martin Hutchings	Retrospective	Glofitamab	–	–	–
Burhan Ferhanoglu	Retrospective (compassionate use)	Glofitamab	23%	28.6%	8.6%

*CRS* cytokine release syndrome, *AEs* adverse events

In another retrospective study, Anna Dodero [5] enrolled 51 patients who did not respond to axi-cel (n = 17) or tisa-cel (n = 34) therapies. Failure after CAR T-cell treatment occurred at a median of 49 days, with 9 patients (18%) experiencing early progression (≤ 30 days). In total, 22 (43%) patients were enrolled in a clinical trial, some of which were given glofitamab (n = 18), while others were adminstered loncastuximab + ibrutinib (n = 4). The ORR and CR were 68% and 38% in patients receiving glofitamab, respectively.

Martin Hutchings [6] presented data related to the CR in end-of-treatment patients with R/R large B-cell lymphoma. A total of 61 patients who received glofitamab were in CR by the end-of-treatment. The median number of prior therapeutic approaches was 3 (range, 2–9), with 61% of patients having received ≥ 3 prior therapies. The ORR and CR were 84% and 56% after 12 months post-end-of-treatment, respectively. This study is one of the first to describe sustained responses lasting after the end of the therapy.

In another real-world study, Burhan Ferhanoglu [7] retrospectively analyzed 42 patients who received glofitamab via compassionate use in Turkey. The median number of prior therapeutic approaches for the patients was 4 (range 3‒6). The ORR and CR were 28.5% and 19%, respectively. The median overall survival was 7 months (95% CI 4.02‒10.03). Observed reduced response rates may be related to an increased number of patients being refractory to the first lines of therapy.

In R/R lymphoma, a reported phase I, dose-escalation preclinical study suggested a strong rationale for combination of RG6333, which is a CD19-targeted affinity-optimized CD28 bispecific antibody, with glofitamab to deepen and prolong treatment responses [8]. Optimal scheduling, including alternation of costimulatory bispecific antibodies suggests a powerful off-the-shelf T cell redirection approach as a potential alternative to CAR-T cell therapies. Given the encouraging long-term data, it seems probable that glofitamab may be used prior to CAR-T due to the off-the-shelf availability of glofitamab treatment as well as the lack of requirement for specialized centers to administer it.

## Data Availability

The material supporting the conclusion of this study has been included within the article.

## Abbreviations

ASH: American Society of Hematology
DLBCL: Diffuse large B cell lymphoma
CRS: Cytokine release syndrome
CR: Complete response
ORR: Overall response rate
PFS: Progression free survival
OS: Overall survival
